# Complete Genome Sequence of *Xanthomonas* Phage Pagan

**DOI:** 10.1128/MRA.01031-19

**Published:** 2019-09-26

**Authors:** Marielle Russo, Tram Le, Russell Moreland, Carlos F. Gonzalez, Mei Liu, Jolene Ramsey

**Affiliations:** a Center for Phage Technology, Texas A&M University, College Station, Texas, USA; DOE Joint Genome Institute

## Abstract

The T7-like podophage Pagan infects Xanthomonas sp. strain ATCC PTA-13101, which was isolated from rice. The 44-kbp Pagan genome contains direct terminal repeats and contains 59 genes, 27 of which have a predicted function. Pagan is most closely related to *Xanthomonas* phage phi Xc10 and Xylella phage Prado.

## ANNOUNCEMENT

The genus *Xanthomonas* consists of many diverse species of Gram-negative, rod-shaped phytopathogenic gammaproteobacteria (1). Members of the genus *Xanthomonas* are ubiquitous and cause a variety of diseases in many plants, including bacterial spot disease of tomatoes and peppers and bacterial leaf blight of rice (2, 3). The resulting diseased crop is unusable, which leads to economically important crop yield loss, which is as high as 50% in rice cultivation (2). *Xanthomonas* and *Xylella* are closely related genera, and Xylella fastidiosa causes Pierce’s disease of grapevines (4). Phage-based therapeutics utilizing isolated virulent *Xylella* phages have exhibited significant potential for treating Pierce’s disease (5).

Phage Pagan was isolated from filtered (pore size, 0.2 μm), mixed freshwater collected in Vidor, TX, and infects the rice-associated *Xanthomonas* sp. strain ATCC PTA-13101. The host was grown aerobically at 28°C in tryptone nutrient broth/agar (BD), and phages were propagated via the soft agar overlay method described previously (6, 7). Full genomic DNA was purified following the shotgun library preparation protocol described by Summer (8), prepared as Illumina TruSeq libraries with the Nano low-throughput kit, and sequenced on an Illumina MiSeq instrument with paired-end 250-bp reads using v2 500-cycle chemistry. The quality of the 28,419 total sequence reads from the index containing the phage genome was evaluated using FastQC (www.bioinformatics.babraham.ac.uk/projects/fastqc). Sequence reads were then trimmed using the FASTX-Toolkit v0.0.14 (http://hannonlab.cshl.edu/fastx_toolkit/). The genome was assembled through SPAdes v3.5.0 (using default parameters) with a sequencing coverage of 6.3-fold (9). Next, the genome was closed by PCR amplification off the contig ends (forward primer, 5′-GGTAGGTGATGTGGCAGTC-3′; reverse primer, 5′-GTGTTCAACGTAGGTGAGAAGG-3′) and subsequent Sanger sequencing. Genome annotation and bioinformatic analysis were conducted through software tools available in the Center for Phage Technology Galaxy and Web Apollo instances (https://cpt.tamu.edu/galaxy-pub/) (10, 11). Structural annotation of the genome was accomplished using Glimmer v3.0 and MetaGeneAnnotator v1.0 and with ARAGORN v2.36 for tRNAs (12–14). Gene function was then predicted using default settings for conserved domain searches with InterProScan v5.33-72 and sequence similarity searches of the NCBI nonredundant or UniProtKB Swiss-Prot/TrEMBL databases with BLAST v2.2.31 at a 0.001 maximum expectation value (15–17). Likely transmembrane domains were identified by TMHMM v2.0, and lipoboxes were identified by LipoP v1.0 (18, 19). Rho-independent termination sites were annotated with TransTermHP v2.09 (20). progressiveMauve v2.4.0 was used to calculate genome-wide DNA sequence similarity (21). Phage morphology was assessed by negatively staining samples with 2% (wt/vol) uranyl acetate and viewing through transmission electron microscopy at the Texas A&M Microscopy and Imaging Center (22).

The 44,448-bp podophage Pagan genome has a coding density of 96.95% and contains 59 predicted genes but no tRNAs. The genome of Pagan displayed a G+C content of 62.3%, near the host *Xanthomonas* genome average of 64% G+C content (4). The contig was reopened at the direct terminal repeat boundary predicted by PhageTerm and that is syntenic with T7-like phages (23). Pagan is most closely related to *Xanthomonas* phage phi Xc10 (GenBank accession number MF375456) and *Xylella* phage Prado (accession number KF626667), with 93.46% and 69.55% nucleotide sequence identity and 53 and 48 shared proteins, respectively (24). These similarities contributed to lysis gene identification, including that of a class II pinholin, signal-arrest-release (SAR) endolysin, and overlapping spanin genes.

### Data availability.

The genome sequence and associated data for phage Pagan were deposited under GenBank accession number MK903278, BioProject accession number PRJNA222858, SRA accession number SRR8892144, and BioSample accession number SAMN11408680.
